# Intensive care treatments associated with favorable discharge outcomes in Argentine children with severe traumatic brain injury: *For the South American Guideline Adherence Group*

**DOI:** 10.1371/journal.pone.0189296

**Published:** 2017-12-15

**Authors:** Monica S. Vavilala, Silvia B. Lujan, Qian Qiu, Michael J. Bell, Nicolás M. Ballarini, Nahuel Guadagnoli, María Alejandra Depetris, Gabriela A. Faguaga, Gloria M. Baggio, Leonardo O. Busso, Mirta E. García, Osvaldo R. González Carrillo, Paula L. Medici, Silvia S. Sáenz, Elida E. Vanella, Carly K. Farr, Gustavo J. Petroni

**Affiliations:** 1 Anesthesiology and Pain Medicine, Harborview Injury Prevention and Research Center, University of Washington, Seattle, Washington, United States of America; 2 Centro de Informática e Investigación Clínica, Rosario, Santa Fe, Argentina; 3 Hospital de emergencias Dr. Clemente Álvarez, Rosario, Santa Fe, Argentina; 4 Pediatrics, Critical Care Medicine, Children’s National Hospital, Washington D.C., United States of America; 5 Hospital de Niños Víctor J Vilela, Rosario, Santa Fe, Argentina; 6 Hospital El Cruce. Ezpeleta Oeste, Buenos Aires, Argentina; 7 Hospital de Niños Sor María Ludovica, La Plata, Buenos Aires, Argentina; 8 Hospital de Niños Dr. Orlando Alassia, Santa Fe, Santa Fe, Argentina; 9 Hospital Interzonal Especializado Materno Infantil Dr. Vitorio Tetamanti, Mar del Plata, Buenos Aires, Argentina; 10 Hospital de Niños de la Santísima Trinidad, Córdoba, Cordoba, Argentina; 11 Hospital Pediátrico Dr. Humberto Notti, Mendoza, Mendoza, Argentina; Public Library of Science, FRANCE

## Abstract

**Objective:**

Little is known about the critical care management of children with traumatic brain injury (TBI) in low middle income countries. We aimed to identify indicators of intensive care unit (ICU) treatments associated with favorable outcomes in Argentine children with severe TBI.

**Methods:**

We conducted a secondary analysis of data from patients previously enrolled in a prospective seven center study of children with severe TBI who were admitted to an ICU in one of the seven study centers. Severe TBI was defined by head AIS ≥ 3, head CT with traumatic lesion, and admission GCS < 9. Seven indicators of best practice TBI care were examined. The primary outcome was discharge Pediatric Cerebral Performance Category Scale [PCPC] and Pediatric Overall Performance category Scale [POPC]. We also examined variation in ICU care and in-patient mortality.

**Results:**

Of the 117 children, 67% were male and 7.5 (4.3) years on average, 92% had isolated TBI. Hypotension (54%) was more common than hypoxia (28%) and clinical or radiographic signs of high intracranial pressure (ICP) were observed in 92%. Yet, ICP monitoring occurred in 60% and hyperosmolar therapy was used in only 36%. Adherence to indicators of best TBI practice ranged from 55.6% to 83.7% across the seven centers and adherence was associated with favorable discharge PCPC (aRR 0.98; 95% CI [0.96, 0.99]), and POPC (aRR 0.98; 95% CI [0.96, 0.99]). Compared to patients whose adherence rates were below 65%, patients whose adherence rates were higher between 75%-100% had better discharge PCPC (aRR 0.28; 95% CI [0.10, 0.83]) and POPC (aRR 0.32; 95% CI [0.15, 0.73]. Two indicators were associated with favorable discharge PCPC: Avoidance of hypoxia (aRR 0.46; 95% CI [0.23, 0.93]), and Nutrition started in 72 hours (aRR 0.45; 95% CI [0.21, 0.99]). Avoiding hypoxia was also associated with favorable discharge POPC (aRR 0.47; 95% CI [0.22, 0.99]).

**Conclusion:**

There is variation in Argentine ICU practice in the care of children with severe TBI. Second insults are common and hyperosmolar therapy use is uncommon. Adherence to best practice TBI care by avoiding hypoxia and providing timely nutrition were associated with significantly favorable discharge outcomes. Implementing strategies that prevent hypoxia and facilitate early nutrition in the ICUs are urgently needed to improve pediatric TBI outcomes.

## Introduction

An estimated 3 million children worldwide suffer from traumatic brain injury (TBI) annually [1]. Argentina has higher incidence of TBI than the United States, and compared to the international average, the incidence of pediatric TBI is 2.97 times higher in Latin America [2,3]. According to the 2015 Argentine Ministry of Health report, accident-related deaths accounted for 5.6% and 27.5% of fatalities for children aged 0 to 4 and 5 to 14 years of age, respectively [4]. While a plethora of statistics is available for western nations such as the United States, pediatric TBI data from South America are limited.

Primary TBI ensues from the mechanical damage to the brain at the time of trauma, and secondary injury results from a host of complex hemodynamic, metabolic, inflammatory and/or excitotoxicity processes [5–9]. Whereas the primary injury is preventable from a public health perspective, it is not preventable by the clinicians who encounter these patients in a medical setting. However, clinicians can provide best practice interventions to prevent second insults such as hypotension, hypoxia to prevent secondary injury [9–13]). In 2012, The Brain Trauma Foundation released the second edition of the evidence-based Guidelines for the Acute Medical Management of Severe Traumatic Brain Injury in Infants, Children, and Adolescents [13]. Guideline recommendations specify certain intensive care unit (ICU) monitoring and therapeutic approach thresholds such as use of intracranial pressure monitoring, treatment intervention at a threshold ICP, minimum cerebral perfusion pressure (CPP), advanced neuromonitoring, and nutrition associated with discharge survival and favorable outcomes after TBI [13]. Adherence to the pediatric TBI guidelines across 5 leading U.S. pediatric trauma centers has been associated with favorable discharge survival and Glasgow outcomes score (GOS). In particular, provision of early nutritional support, avoidance of hyperventilation, and maintenance of CPP were found to be protective, even adjustment for injury severity. Graves et al suggests that greater guideline adherence was not associated with higher cost [14].

While intended for worldwide use and not limited to adoption in the western world, the TBI guidelines for severe pediatric TBI were derived largely from data obtained in the United States and Europe. Additionally, some guideline topics may be beyond the scope of usual clinical practice in South American countries such as Argentina. However, previous work suggests that examining the care of pediatric TBI patients from Argentina is valuable and enhances our common understanding of the relationship between the quality of TBI care provided and outcomes. We recently benchmarked prehospital and emergency care of children with all TBI severities who received care at seven Argentine trauma centers and showed that many critically injured children with TBI do not receive timely or best practice prehospital care and that that longer transport time is associated with poor discharge outcomes [15]. We have also shown that there is generalizability of the five World Health Organization/Organization Mondiale de la Santé TBI prognostic predictors in both high income countries and LMICs [2–3, 16–19]. However, these studies did not examine specifics of ICU critical care. These findings suggest there may be commonalities in TBI pathophysiology patterns, and in potential treatments for patients with severe TBI, and applicability for the severe Pediatric TBI guidelines in countries like Argentina which often have comparable ICU infrastructure. Levering our prior work, we aimed to understand the role and value of select intensive care treatments recommended in the TBI guidelines in Argentina.

## Materials and methods

### Overview

As previously described, this is an international collaboration of seven Argentine pediatric trauma centers which formed a network to study TBI care and outcomes (15). The present study is a secondary analysis of prospectively collected data from this network. All seven study sites have Federal Wide Assurance approval. Hospitals and their respective ethics committees are: 1) Hospital de Niños Víctor J. Vilela, Rosario, Argentina (Secretaria Salud Pública Municipalidad de Rosario); 2) Hospital El Cruce, Florencio Varela, Argentina (Hospital Alta Complejidad El Cruce Dr. Nestor Carlos Kirchner); 3) SAMICHospital de Niños Sor María Ludovica, La Plata, Argentina (Hosp de Ninos de la Plata Sup Sor Maria Ludovica IRB; 4) Hospital de Niños “Dr. Orlando Alassia”, Santa Fe, Argentina (Comité de Etica en Investigación); 5) Hospital J. B. IturraspeHospital Interzonal Especializado Materno Infantil Dr. Vitorio Tetamanti, Mar del Plata, Argentina (Consejo Institucional de Revisión de Estudios de Investigación); 6_ Hospital de Niños de la Santísima Trinidad, Córdoba, Argentina (Comité Insitucional de Etica de la Investigacion en salud del Niño y del Adulto); and 7) Polo HospitalarioHospital Pediátrico Dr. Humberto Notti, Mendoza, Argentina (Hospital Central IRB).

The study and the consent process were approved by the local ethical committees which were responsible for study oversight and the consent/assent process in Spanish. Consent was obtained in writing from the next of kin, caretakers, or guardians on behalf of the minors/children enrolled in our study, and written child assent was attempted and obtained when possible for all children age 7 years and older). We documented consent processes and consents and stored consent forms in a secure file accessible only to investigators.

### Study center characteristics

All seven pediatric study centers are designated as high complexity hospitals and are level 1 trauma centers. All Ped ICUs provide multidisciplinary care to patients with serious or complex diseases, including medical, surgical or traumatic conditions. Level 1 pediatric trauma center ICUs are located in pediatric hospitals or general hospitals of high complexity. These pediatric ICUS provide pediatric specialties and subspecialties, both medical and surgical, that contribute to meeting the physiological and emotional needs of critically ill children.^(3)^ Specifically, all seven trauma hospitals have capacity and are approved to provide intracranial pressure (ICP) monitoring, have a TBI champion, and are public hospitals which are located in large urban areas. All seven centers have computed tomography scanning capacity, a pediatric intensive care unit with 8–24 beds, and 24 hour neurosurgeon availability. Typically, all children with severe TBI patients receive a head CT scan within first 24 hours of injury. The current healthcare system does not sustain a complex, expensive rehabilitation program. Survivors are discharged to their families. The burden of illness is particularly poignant for pediatric TBI patients, as these children cannot return to school or engage in productive activities in their communities. In most of patient, in the post-acute care the family provides home-based care. If ICP monitoring was not performed, clinicians used CT findings to diagnose herniation, per local practice.

### Data sources and data collection

Data sources were a prospective study, which evaluated the effectiveness and sustainability of a new post TBI discharge trauma care protocol among 308 children who survived to discharge with TBI and of 58 patients who underwent local hospital’s standard of care.

The instrument and data collection variables were developed according to the National Institute of Neurological Disorders and Stroke Common Data Elements project [20]. Data were collected on paper and then entered into a password protected web based database. Data cleaning was in real time by Argentine data managers. Site visits were conducted every 3 months to ensure data collection. For this study, data were analyzed by QQ, and MSV. All patient evaluation, data collection and entry, quality control, and local administration were conducted by this group.

### Study population

Eligible participants for this analysis who were patients between 0–18 years of age admitted to one of the center ICUs with a diagnosis of severe TBI (admission Glasgow Coma scale score [GCS] ≤ 8 and traumatic injury on head CT scan on admission, and head AIS > 3) and were followed up to hospital discharge between August 2011 to June 2013. Since this study focuses on ICU care and subsequent outcomes, participants who died in the ED were excluded.

### Measures of ICU TBI care

We examined seven ICU clinical indicators representing measures of adherence to best TBI care practice, in accordance with TBI Guidelines (Table 1) and scope of typical local practice. Consequently, adherence to some guideline indicators, such as advanced neuromonitoring, was not included in the calculation of guideline adherence. The number and type of indicators was determined *a priori* by the study group based on previous work, local context and the feasibility of reliably interpreting these data collected as part of our prior work in Argentina. Some indicators were deemed to be conditional on occurrence and when denominator data were available, were examined as such [12,20]. Examples of conditional clinical indicators are hypertonic saline or mannitol administration for high ICP. The definition of high ICP was based on presence of any of the following: ICP > 20 mmHg; documented herniation; mannitol or hypertonic saline were administered, Marshall Classification Diffuse Injury III or IV, or abnormal pupillary examination consistent with concern for herniation. Table 1 describes the list of ICU indicators used to determine TBI guideline adherence during ICU care. Based on consistency of available data and indicators, no systolic hypotension (all systolic blood pressure ≥ SBP ± 2*age), no hypoxia (all SaO_2_ ≥ 90% or all PaO_2_ ≥ 60mmHg), use of antiepileptic drugs, no use of steroids, and starting nutrition within 72 hours after ICU admission [12, 20] were examined. For each patient indicator, a value of 0 was assigned to lack of adherence and a value of 1 was assigned for adherence. Both direct and indirect transfers were included. For conditional indicators such as treatment of high ICP with hypertonic saline or mannitol, patients without clinical or radiographic evidence of high ICP were excluded from both numerator and denominator when calculating adherence rates. We calculated a best practice adherence rate for each patient using the sum of the numbers of indicators to which care was provided, divided by the sum of relevant indicators for that patient. Mean overall ICU adherence rates for all patients at all study centers were also determined.

**Table 1:** Scheme for examining traumatic brain injury guideline adherence indicators in the intensive care unit (ICU)*.

Indicators	Definition and coding	ICU Indicator
		(n = 7)
1. Hypertonic saline or Mannitol for high ICP	0 = Neither hypertonic saline nor Mannitol provided for patient with high ICP	X
	1 = Hypertonic saline or Mannitol provided for patient with high ICP	
*Condition*: *High ICP*	2 = No high ICP	
2. Systolic hypotension avoided	0 = No	X
	1 = Yes	
	2 = Unknown	
3. ICP monitored	0 = No ICP monitored	X
	1 = ICP monitored	
	2 = Unknown	
4. Hypoxia avoided	0 = No	X
	1 = Yes	
	2 = Unknown	
5. Prophylactic antiepileptic drugs used to prevent early seizures	0 = No prophylactic antiepileptic drugs used	X
	1 = Prophylactic antiepileptic drugs used	
6. Steroids not used	0 = Steroids administered	X
	1 = Steroids not administered	
	2 = Unknown	
7. Nutrition started in 72 hours after ICU admission	0 = Nutrition not started	X
	0 = Nutrition start >72 hours after ICU admission	
	1 = Nutrition start ≤ 72 hours after ICU admission	
	2 = Unknown nutrition time	

*ICU guidelines recommended for patients with severe TBI

### Outcomes

The primary outcomes were discharge Pediatric Cerebral Performance Category Scale (PCPC) and Pediatric Overall Performance category Scale (POPC); both are scored between one and six where score 1 = good, 2 = mild disability, 3 = moderate disability, 4 = severe disability, 5 = vegetative state, and 6 = death. A dichotomous measure of poor outcome (severe-vegetative and death) vs. favorable (normal, mild-moderate disability) was used for both PCPC and POPC (16–19). We also examined variation in ICU adherence to seven ICU TBI treatments.

### Statistical analyses

Patient demographic, clinical and transport details were described across seven centers. Data are presented as mean (standard errors) or median (interquartile range) for continuous variables and count (percentage) for categorical variables. Patient and injury level characteristics were examined in bivariate analyses by discharge PCPC and POPC, using Student’s t-test for continuous variables and χ2 tests for categorical variables. Adherence rates for each of seven ICU indicators were compared across seven centers and mean ICU adherence rates were calculated for each center.

Multivariate modified Poisson regression analyses (with a robust error variance to estimate the relative risk) were used to examine the effect of ICU adherence on PCPC and POPC at hospital discharge. To examine the effect of individual indicator, for both PCPC and POPC, ICU adherence indicators were first examined singly adjusting for clinical confounders. Adherence indicators with a p-value of less than 0.2 were retained and entered into a final model. Stepwise selection was used in final model to successively dropping indicators with a p-value greater than 0.05, starting with the indicator with the highest p-values until all the remaining adherence indicators were significant with a p-value less than 0.05. All multiple regressions adjusted for age, sex, maximum head AIS, maximum non-head AIS, and motor GCS at admission. We used GCS motor score on admission since this measure of severity^12^, and this information was available for us to examine. GCS motor at admission is classified as: 1 = No motor response; 2 = Extension to pain; 3 = Flexion to pain; 4 = Withdrawal from pain; 5 = Localizing pain; 6 = Obeys Commands). All analyses controlled for clustering effect within each trauma center. Regression coefficients or adjusted relative risks and related 95% CIs were reported for each model. Statistical significance was defined with a p value less than 0.05. Stata MP 13.1 (Stata Corporation, College Station, TX) was used for all analyses.

## Results

### Clinical characteristics

Data from these patients have been included in our prior analysis of prehospital and emergency care of children with TBI at these centers but there was no formal analysis of children with severe TBI (15). This study focuses on children with severe TBI. Respectively, Tables 2 and 3 describes clinical and outcome characteristics for the cohort and by each study center. Of the 117 children with severe TBI who were identified with ICU stay during hospitalization, patients were 7.5 (4.3) years, mostly (66.7%) male, 83% received care at an outside facility prior to definitive care admission, and 92% had isolated TBI. The majority (58.1%) were injured during traffic accidents, followed by falls from height (17.1%). The most common admission GCS motor score was 1 = no motor response (56.4%) and the most common (28.2%) head abbreviated injury severity score was 3 = serious. Injury severity score was 18.5 (11.6). Cerebral contusions were the most common head CT diagnosis (34.2%) and 31.6% of TBI patients underwent surgery. Mortality and disability were examined for the whole cohort, including patients with and without invasive ICP monitoring. High ICP was defined as evidence by either invasive ICP monitoring, or clinical or radiological signs. Three (2.6%) patients died and 42.8% had some form of discharge disability. S1 Table shows detailed clinical characteristics of the 117 children with TBI across seven study centers by discharge outcomes (univariate associations).

**Table 2:** Clinical characteristics of 117 children with severe traumatic brain injury admitted intensive care unit (ICU) across seven study centers.

	Total	*Center 1*	*Center 2*	*Center 3*	*Center 4*	*Center 5*	*Center 6*	*Center 7*
	*n = 117*	*n = 15*	*n = 34*	*n = 14*	*n = 25*	*n = 13*	*n = 9*	*n = 7*
	N(%)	*N(%)*	*N(%)*	*N(%)*	*N(%)*	*N(%)*	*N(%)*	*N(%)*
**Age (years)** mean[SD]	7.5[4.3]	7.8[4.6]	7.5[4.4]	9.2[4.5]	6.4[4.7]	7.4[4.1]	7.9[2.5]	8.0[4.4]
**Sex**								
Male	78 (66.7)	11 (73.3)	22 (64.7)	10 (71.4)	14 (56.0)	9 (69.2)	6 (66.7)	6 (85.7)
**Direct transfer from scene**								
Yes	20 (17.1)	3 (20.0)	6 (17.7)	2 (14.3)	1 (4.0)	3 (23.1)	2 (22.2)	3 (42.9)
** Injury mechanism**								
Traffic accident	68 (58.1)	11 (73.3)	20 (58.8)	8 (57.1)	12 (48.0)	6 (46.2)	4 (44.4)	7 (100.0)
Fall from height	20 (17.1)	0 (0.0)	2 (5.9)	2 (14.3)	6 (24.0)	5 (38.5)	5 (55.6)	0 (0.0)
Fall from own height	3 (2.6)	0 (0.0)	1 (2.9)	1 (7.1)	1 (4.0)	0 (0.0)	0 (0.0)	0 (0.0)
Strike	11 (9.4)	2 (13.3)	5 (14.7)	0 (0.0)	4 (16.0)	0 (0.0)	0 (0.0)	0 (0.0)
Gunshot wound	5 (4.3)	1 (6.7)	1 (2.9)	2 (14.3)	0 (0.0)	1 (7.7)	0 (0.0)	0 (0.0)
Other / Unknown	10 (8.6)	1 (6.7)	5 (14.7)	1 (7.1)	2 (8.0)	1 (7.7)	0 (0.0)	0 (0.0)
**Injury circumstance**								
Child abuse	1 (0.9)	0 (0.0)	0 (0.0)	0 (0.0)	1 (4.0)	0 (0.0)	0 (0.0)	0 (0.0)
Intentional(no child abuse)	4 (3.4)	1 (6.7)	1 (2.9)	1 (7.1)	0 90.0)	1 (7.7)	0 (0.0)	0 (0.0)
Accidental	111 (94.9)	14 (93.3)	32 (94.1)	13 (92.9)	24 (96.0)	12 (92.3)	9 (100.0)	7 (100.0)
Other / Unknown	1 (0.9)	0 (0.0)	1 (2.9)	0 (0.0)	0 (0.0)	0 (0.0)	0 (0.0)	0 (0.0)
**Glasgow coma scale score (admit motor)**								
1	66 (56.4)	7 (46.7)	21 (61.8)	5 (35.7)	16 (64.0)	10 (76.9)	6 (66.7)	1 (14.3)
2	7 (6.0)	1 (6.7)	2 (5.9)	2 (14.3)	1 (4.0)	0 (0.0)	0 (0.0)	1 (14.3)
3	2 (1.7)	1 (6.7)	0 (0.0)	1 (7.1)	0.0 (0.0)	0 (0.0)	0 (0.0)	0 (0.0)
4	18 (15.4)	2 (13.3)	2 (5.9)	5 (35.7)	3 (12.0)	2 (15.4)	1 (11.1)	3 (42.9)
5	10 (8.6)	1 (6.7)	6 (17.7)	0 (0.0)	1 (4.0)	0 (0.0)	0 (0.0)	2 (28.6)
6	3 (2.6)	0 (0.0)	1 (2.9)	0 (0.0)	1 (4.0)	1 (7.7)	0 (0.0)	0 (0.0)
Unknown	11 (9.4)	3 (20.0)	2 (5.9)	1 (7.1)	3 (12.0)	0 (0.0)	2 (22.2)	0 (0.0)
Total admission Glasgow coma scale score	3.9 (1.7)	3.8 (1.7)	3.9 (1.9)	3.9 (1.4)	3.4 (1.3)	3.6 (1.6)	4.1 (2.2)	6.0 (1.8)
**Head abbreviated injury severity score (AIS)**								
1	3 (2.6)	0 (0.0)	0 (0.0)	0 (0.0)	0 (0.0)	0 (0.0)	3 (33.3)	0 (0.0)
2	21 (18.0)	2 (13.3)	8 (23.5)	3 (21.4)	2 (8.0)	5 (38.5)	1 (11.1)	0 (0.0)
3	33 (28.2)	4 (26.7)	14 (41.2)	4 (28.6)	2 (8.0)	6 (46.2)	1 (11.1)	2 (28.6)
4	30 (25.6)	3 (20.0)	7 (20.6)	1 (7.1)	12 (48.0)	2 (15.4)	2 (22.2)	3 (42.9)
5	29 (24.8)	6 (40.0)	5 (14.7)	6 (42.9)	8 (32.0)	0 (0.0)	2 (22.2)	2 (28.6)
6	1 (0.9)	0 (0.0)	0 (0.0)	0 (0.0)	1 (4.0)	0 (0.0)	0 (0.0)	0 (0.0)
**Injury severity score** mean[SD]	18.5[11.6]	21[11.4]	16[10.1]	22.3[14.9]	21.3[12.8]	11.4[6.0]	14.7[10.0]	25.3[9.7]
** **								
**Non-head MAXAIS**								
0	55 (47.0)	8 (53.3)	12 (35.3)	5 (35.7)	18 (72.0)	7 (53.9)	3 (33.3)	2 (28.6)
1	17 (14.5)	3 (20.0)	8 (23.5)	2 (14.3)	1 (4.0)	0 (0.0)	2 (22.2)	1 (14.3)
2	18 (15.4)	1 (6.7)	6 (17.7)	2 (14.3)	3 (12.0)	4 (30.8)	2 (22.2)	0 (0.0)
3	20 (17.1)	1 (6.7)	7 (20.6)	4 (28.6)	3 (12.0)	2 (15.4)	1 (11.1)	2 (28.6)
4	5 (4.3)	1 (6.7)	0 (0.0)	1 (7.1)	0 (0.0)	0 (0.0)	1 (11.1)	2 (28.6)
5	2 (1.7)	1 (6.7)	1 (2.9)	0 (0.0)	0 (0.0)	0 (0.0)	0 (0.0)	0 (0.0)
**Hospital stay (days)** mean[SD]	20.0[18.7]	22.3[22.8]	20.4[19.3]	25.1[20.5]	16.3[15.0]	9.7[4.9]	31.7[26.9]	20.9[9.8]
**Extracranial injury**								
No	108 (92.3)	14 (93.3)	30 (88.2)	12 (85.7)	25 (100.0)	11 (84.6)	9 (100.0)	7 (100.0)
Yes	9 (7.7)	1 (6.7)	4 (11.8)	2 (14.3)	0 (0.0)	2 (15.4)	0 (0.0)	0 (0.0)
**Injury location**								
Head/Face	117 (100.0)	15 (100.0)	34 (100.0)	14 (100.0)	25 (100.0)	13 (100.0)	9 (100.0)	9 (100.0)
Neck	6 (5.1)	1 (6.7)	2 (5.9)	2 (14.3)	0 (0.0)	0 (0.0)	1 (11.1)	0 (0.0)
Thorax	27 (23.1)	2 (13.3)	10 (29.4)	6 (42.9)	3 (12.0)	4 (30.8)	1 (11.1)	1 (14.3)
Abdomen	18 (14.4)	3 (20.0)	4 (14.7)	5 (35.7)	2 (8.0)	1 (7.7)	2 (22.2)	0 (0.0)
Spine	4 (3.4)	0 (0.0)	2 (5.9)	2 (14.3)	0 (0.0)	0 (0.0)	0 (0.0)	0 (0.0)
Extremities	31 (26.5)	5 (33.3)	11 (32.4)	5 (35.7)	5 (20.0)	2 (15.4)	0 (0.0)	3 (42.9)
External and Other	6 (5.1)	1 (6.7)	3 (8.8)	2 (14.3)	0 (0.0)	0 (0.0)	0 (0.0)	0 (0.0)
**All head computed tomography diagnoses**								
Epidural hematoma	23 (17.0)	4 (26.7)	4 (11.8)	2 (14.3)	9 (36.0)	2 (22.2)	2 (22.2)	0 (0.0)
Subdural hematoma	15 (12.8)	2 (13.3)	1 (2.9)	5 (35.7)	2 (8.0)	2 (15.4)	2 (22.2)	1 (14.3)
Subarachnoid hemorrhage	9 (7.7)	2 (13.3)	3 (8.8)	0 (0.0)	3 (12.0)	0 (0.0)	0 (0.0)	1 (14.3)
Intracerebral hemorrhage	7 (6.0)	1 (6.7)	0 (0.0)	2 (14.3)	2 (8.0)	0 (0.0)	1 (11.1)	1 (14.3)
Intraventricular hemorrhage	8 (6.8)	1 (6.7)	2 (5.9)	0 (0.0)	4 (16.0)	0 90.0)	0 (0.0)	1 (14.3)
Contusion	40 (34.2)	10 (66.7)	10 (29.4)	4 (28.6)	5 (20.0)	2 (15.4)	4 (44.4)	5 (71.4)
**Any surgery**								
No	80 (68.4)	9 (60.0)	27 (79.4)	10 (71.4)	12 (48.0)	10 (76.9)	6 (66.7)	6 (85.7)
Yes	37 (31.6)	6 (40.0)	7 (20.6)	4 (28.6)	13 (52.0)	3 (23.1)	3 (33.3)	1 (14.3)
**Decompressive craniectomy**								
No	102 (87.2)	13 (86.7)	30 (88.2)	10 (71.4)	22 (88.0)	13 (100.0)	7 (77.8)	7 (100.0)
Yes	14 (12.0)	2 (13.3)	4 (11.8)	4 (28.6)	3 (12.0)	0 (0.0)	1 (11.1)	0 (0.0)
NA / Missing	1 (0.9)	0 (0.0)	0 (0.0)	0 (0.00	0 (0.0)	0 (0.0)	1 (11.1)	0 (0.0)

**Table 3:** Outcomes of 117 children with severe traumatic brain injury admitted to seven study centers.

	Total	*Center 1*	*Center 2*	*Center 3*	*Center 4*	*Center 5*	*Center 6*	*Center 7*
	n = 117	*n = 15*	*n = 34*	*n = 14*	*n = 25*	*n = 13*	*n = 9*	*n = 7*
** **	N(%)	*N(%)*	*N(%)*	*N(%)*	*N(%)*	*N(%)*	*N(%)*	*N(%)*
**Discharge disposition**								
Home w/o homecare	106 (90.6)	13 (86.7)	31 (91.1)	13 (92.9)	20 (80.0)	13 (100.0)	9 (100.0)	7 (100.0)
Home with homecare	2 (1.7)	2 (13.3)	0 (0.0)	0 (0.0)	0 (0.0)	0 (0.0)	0 (0.0)	0 (0.0)
Other hospital	6 (5.1)	0 (0.0)	2 95.9)	1 (7.1)	3 (12.0)	0 (0.0)	0 (0.0)	0 (0.0)
Death	3 (2.6)	0 (0.0)	1 (2.9)	0 (0.0)	2 (8.0)	0 (0.0)	0 (0.0)	0 (0.0)
** **								
**Pediatric Cerebral Performance category Scale (PCPC) at Hospital discharge**								
Normal	62 (53.0)	7 (46.7)	19 (55.9)	6 (42.9)	13 (52.0)	11 (84.6)	3 (33.3)	3 (42.9)
Mild disability	21 (18.0)	4 (26.7)	6 (17.7)	2 (14.3)	1 (4.0)	2 (15.4)	4 (44.4)	2 (28.6)
Moderate disability	12 (10.3)	1 (6.7)	7 (20.6)	1 (7.1)	2 (8.0)	0 (0.0)	0 (0.0)	1 (14.3)
Severe disability	17 (14.5)	2 (13.3)	1 (2.9)	4 (28.6)	7 (28.0)	0 (0.0)	2 (22.2)	1 (14.3)
Coma or vegetative state	2 (1.7)	1 (6.7)	0 (0.0)	1 (7.1)	0 (0.0)	0 (0.0)	0 (0.0)	0) 0.0)
Brain death	3 (2.6)	0 (0.0)	1 (2.9)	0 (0.0)	2 (8.0)	0 (0.0)	0 (0.0)	0 (0.0)
** **								
**Pediatric Overall Performance Category Scale (POPC) at Hospital discharge**								
Good overall performance	58 (50.0)	8 (53.3)	15 (44.1)	5 (35.7)	12 (48.0)	11 (84.6)	4 (44.4)	3 (42.9)
Mild overall performance	24 (20.5)	3 (20.0)	9 (26.5)	3 (21.4)	2 (8.0)	2 (15.4)	3 (33.3)	2 (28.6)
Moderate overall performance	14 (12.0)	1 (6.7)	7 (20.6)	3 (21.4)	2 (8.0)	0 (0.0)	0 (0.0)	1 (14.3)
Severe overall performance	16 (13.7)	2 (13.3)	2 (5.9)	2 (14.3)	7 (28.0)	0 (0.0)	2 (22.2)	1 (14.3)
Coma or vegetative state	2 (1.7)	1 (6.7)	0 (0.0)	1 (7.1)	0 (0.0)	0 (0.0)	0 (0.0)	0 (0.0)
Death	3 (2.6)	0 (0.0)	1 (2.9)	0 (0.0)	2 (8.0)	0 (0.0)	0 (0.0)	0 (0.0)

### Measures of ICU TBI Care

Intensive Care Unit care was examined for 117 patients with severe TBI. Overall ICU guideline adherence was 65.0% with large center variation from 55.6% to 83.7% (Table 4). Nearly 50% of patients experienced systolic hypotension and hypotension was common (24%). Over 30% did not receive antiepileptic medications, and 30% did not receive nutrition within the first 72 hours after ICU admission. Despite over 90% (108 out of 117, regardless of ICP monitoring) of children having clinical or radiographic evidence of high ICP, ICP monitoring occurred in 60% and hyperosmolar therapy use barely exceeded 30%. Steroids were used in less than 5% across all centers.

**Table 4:** Adherence scorecard for severe pediatric traumatic brain injury indicators across seven study centers by intensive care unit (ICU).

* *	* *	*Total (n = 117)*	*Center 1 (n = 15)*	*Center 2 (n = 34)*	*Center 3 (n = 14)*	*Center 4 (n = 25)*	*Center 5 (n = 13)*	*Center 6 (n = 9)*	*Center 7 (n = 7)*
		*%*	*%*	*%*	*%*	*%*	*%*	*%*	*%*
*ICU*	Indicator								
*Indicators*									
Indicator 1	Hypertonic saline or Mannitol for high ICP*	36.1	40.0	27.3	45.5	39.1	10.0	33.3	85.7
	% of patients with high ICP (n = 108) ^a^	92.3	100.0	97.1	78.6	92.0	76.9	100.0	100.0
Indicator 2	Systolic hypotension avoided**	46.0	46.7	42.4	46.2	44.0	50.0	50.0	57.1
Indicator 3	ICP monitored***	60.3	46.7	47.1	57.1	70.8	84.6	44.4	100.0
Indicator 4	Hypoxia avoided****	72.4	50.0	70.6	57.1	72.0	84.6	100.0	100.0
Indicator 5	Prophylactic antiepileptic drugs used to prevent early seizures	69.2	60.0	61.8	85.7	64.0	84.6	66.7	85.7
Indicator 6	Steroids not used*****	96.6	93.3	94.1	100.0	100.0	92.3	100.0	100.0
Indicator 7	Nutrition started in 72 hours after ICU admission******	72.1	53.3	76.5	84.6	83.3	66.7	55.6	57.1
***ICU Adherence rate (%)***		***65*.*0***	***55*.*6***	***59*.*9***	***68*.*2***	***68*.*2***	***70*.*1***	***64*.*6***	***83*.*7***

^a^The indicator includes the entire cohort of patients, with and without invasive ICP monitoring. Data reflect diagnosis of high ICP by either invasive ICP monitoring, clinical or radiological signs.

* ICU hypertonic saline or mannitol for high ICP is a conditional indicator (Center1 [N = 15]; Center2 [N = 33]; Center3 [N = 11]; Center4 [N = 23]; Center5 [N = 10]; Center6 [N = 9]; Center7 [N = 7])

** 6 patients with unknown systolic hypotension avoided was not included in adherence calculation (Center1 [N = 15]; Center2 [N = 33]; Center3 [N = 13]; Center4 [N = 25]; Center5 [N = 12]; Center6 [N = 6]; Center7 [N = 7])

*** 1 patient with unknown ICP monitoring was not included in adherence calculation (Center1 [N = 15]; Center2 [N = 34]; Center3 [N = 14]; Center4 [N = 24]; Center5 [N = 13]; Center6 [N = 9]; Center7 [N = 7])

**** 1 patient with unknown hypoxia avoided was not included in adherence calculation (Center1 [N = 14]; Center2 [N = 34]; Center3 [N = 14]; Center4 [N = 25]; Center5 [N = 13]; Center6 [N = 9]; Center7 [N = 7])

***** 1 patient with unknown usage of steroid was not included in adherence calculation (Center1 [N = 15]; Center2 [N = 34]; Center3 [N = 14]; Center4 [N = 25]; Center5 [N = 13]; Center6 [N = 8]; Center7 [N = 7])

****** 6 patients with unknown nutrition time were not included in adherence calculation (Center1 [N = 15]; Center2 [N = 34]; Center3 [N = 13]; Center4 [N = 24]; Center5 [N = 9]; Center6 [N = 9]; Center7 [N = 7])

Seventy of 117 patients had ICP monitoring. One patient with unknown ICP monitoring status was excluded from adherence analysis for this indicator. Among the 70 patients: 9 (12.9%) patients were identified not having high ICP, and did not receive ICP directed therapy, 26 (37.1%) patients were identified as having high ICP, but did not receive treatment with hypertonic saline or mannitol and 35 (50%) patients were identified with high ICP, and were treated with hypertonic saline or mannitol.

Forty six of 117 patients did not have ICU ICP monitoring. Among those 46 patients, 43 (93.5%) patients were identified as having high ICP based on clinical or radiographic exam but and did not receive treatment of hypertonic saline or mannitol. Three (6.5%) patients were identified with high ICP, and treated with hypertonic saline or mannitol.

### Measures of TBI care and outcomes

Across all study centers, overall ICU indicators of adherence to best practice was associated with favorable discharge PCPC (aRR 0.98; 95% CI [0.96, 0.99]) and POPC (aRR 0.98; 95% CI [0.96, 0.99]). Table 5 shows that compared to patients whose ICU adherence rates were lower, patients with 75%-100% adherence had more favorable outcome for PCPC (aRR 0.28; 95% CI [0.10, 0.83]), and POPC (aRR 0.32; 95% CI [0.15, 0.73]. Table 6 shows that two specific indicators were associated with favorable discharge PCPC: Hypoxia avoided (aRR 0.46; 95% CI [0.23, 0.93]), and nutrition started in 72 hours after ICU admission (aRR 0.45; 95% CI [0.21, 0.99]). Avoiding hypoxia was associated with favorable discharge POPC (aRR 0.47; 95% CI [0.22, 0.99]). Patients who received hypertonic saline or mannitol for high ICP had worse discharge PCPC (aRR 2.15; 95% CI [1.30, 3.56]) and POPC (aRR 2.12; 95% CI [1.15, 2.92]). Only 3 patients died; hence factors associated with mortality were not examined.

**Table 5:** Association between overall ICU adherence rate and by quintile and discharge PCPC and POPC.

ICU	Poor PCPC*		Poor POPC**	
	aRR (95% CI)***		aRR (95% CI)***	
Overall Adherence Rate (%)	0.98 (0.96, 0.99)		0.98 (0.96, 0.99)	
**Adherence Rate Category**				
<65%	N = 23	*Reference*	N = 23	*Reference*
65% to < 75%	N = 28	0.53 (0.16, 1.82)	N = 28	0.32 (0.05, 1.97)
75% to 100%	N = 66	**0.28 (0.10, 0.83)**	N = 66	**0.32 (0.15, 0.73)**

*Dichotomous PCPC (poor outcome = severe-vegetative and brain death vs. favorable outcome = normal, mild-moderate disability)

**Dichotomous POPC (poor outcome = severe-vegetative state and brain death vs. favorable outcome = good-moderate overall performance)

*** Model includes protective indicators. Risk estimates are adjusted relative risk (RR) of adherence rate groups for PCPC and POPC, and adjusted for age, sex, maximum head Abbreviated Injury Score (AIS), maximum non-head AIS and Glasgow Coma Scale score motor

Boldface values denotes statistically significant.

**Table 6:** Association between ICU indicators of adherence to best practice and discharge PCPC and POPC.

Indicators	Poor PCPC*	Poor POPC**
	aRR (95% CI)***	aRR (95% CI)***
**Hypoxia avoided**		
No	Reference	Reference
Yes	**0.46 (0.23, 0.93)**	**0.47 (0.22, 0.99)**
Unknown	Not included in model	Not included in model
**Nutrition started in 72 hours after ICU admission**		
No	*Reference*	---
Yes	**0.45 (0.21, 0.99)**	---
Unknown	0.76 (0.22, 2.58)	---
**Hypertonic saline or Mannitol for high ICP**		
Neither hypertonic saline nor Mannitol used with high ICP	Reference	Reference
Hypertonic saline or Mannitol used with high ICP	**2.15 (1.30, 3.564)**	**2.12 (1.15, 3.92)**
No high ICP	2.06 (0.56, 7.55)	0.84 (0.28, 2.48)

*Dichotomous PCPC (poor outcome = severe-vegetative and brain death vs. favorable outcome = normal, mild-moderate disability)

**Dichotomous POPC (poor outcome = severe-vegetative state and brain death vs. favorable outcome = good-moderate overall performance)

*** Risk estimates are adjusted relative risk (RR) of adherence rate for PCPC and POPC, and adjusted for age, sex, maximum head Abbreviated Injury Score (AIS), maximum non-head AIS and Glasgow Coma Scale score motor

Dashes indicate indicator not in the model. Boldface values denotes statistically significant.

## Discussion

In this study, we aimed to examine the ICU care that Argentine children with severe TBI receive. Problematic observations are that most of these children have high ICP but few receive potentially life-saving monitoring or treatments, and that second insults after TBI are common. There is also large between-center variation in critical care treatments of these children. Yet, higher ICU adherence to best TBI care is beneficial and that select treatments such as avoidance or expedient treatment of hypoxia and timely provision of nutrition are associated with favorable discharge outcomes. This is the first and largest South American study to examine the relationship between ICU care and discharge outcomes in children with severe TBI.

The main findings from South American pediatric trauma centers are similar to that reported in our previous work in five leading pediatric trauma centers in the United States. [12]. While there are certainly differences in injury severity, time to transport and some TBI care measures between the North American and this South American study, there are also some similar findings. First there is large between center variation and an overall TBI guideline adherence rate. Second, avoidance of hypoxia and timely provision of nutrition emerged as protective against poor discharge outcomes, after adjustments for injury severity, regardless of these contextual differences. Additionally, there may be a threshold or dose dose response association between TBI guideline adherence and discharge outcomes. In this study, many children experienced second insults. Published work from North America and Europe similarly report high rates of hypotension and hypoxia, which has sometimes been attributed to the presence of polytrauma [2, 9–12]. Present findings suggest that the origin of these second insults is not necessarily due to extracranial injuries given the fact that over 90% of children in this study had isolated TBI. Therefore, even children with isolated TBI are at significant risk of hypotension and hypoxia. These new regarding second insults occurring in-hospital among children with isolated TBI findings have implications for clinicians who screen and anticipate second insults and who take measures to prevent and expediently treat these adverse TBI specific related sequelae.

The number of children who had either clinical and/or radiographic evidence of high ICP on ICU admission was very high. This finding may reflect injury severity and/or delay in earlier treatments. Despite this staggering occurrence, the use of hyperosmolar therapy at these leading centers is disproportionately low, perhaps being reserved for use in the most severely injured. This is problematic because use of hyperosmolar therapy at the bedside can be life-saving and the lower use may reflect its lack of availability, lack of provider knowledge on how to use these medications, decision to not treat for reasons of futility and/or decision to treat in only certain TBI cases. While only 3 children in this study died in hospital once admitted to the ICU, over 40% were discharged with disability, raising the question as to whether more aggressive use of hyperosmolar therapy would have resulted in better discharge outcomes or whether its use was reserved for the most severe of TBI cases or for those most likely to survive beyond discharge. The latter hypothesis is supported by our finding that use of hypersomolar therapy was associated with worse discharge outcomes.

In this study, over 60% of children underwent ICP monitoring. The recent study which randomized Latin American adults with TBI from similar settings reported no ICP monitoring benefits over neurocritical care [21], and while the strength of study design in Chesnut’s study is obviously superior to the observational design in this study, results are signal that some ICU treatments that require less technology, such as provision of nutrition and ensure oxygenation, are protective, and that that provision of high quality ICU care may be more important than focusing on ICP monitoring alone. There are also other considerations such as timing of ICP monitors and interpretation and reaction to ICP data, which we did not consider due to lack of availability of data. In addition to adhering to best practice TBI care, specific attention to these two treatment factors in the ICU is warranted.

The incidence of abusive head trauma was low, as was the overall mortality rate. It is possible that some centers with low mortality rates will not be able to reduce mortality rates further. Hence, we examined pooled data across these studied sites to better understand the relationship between clinical indicators that are recommended and outcomes in a South American setting. Mortality may also be underappreciated because if patients died in ambulance or at the scene or were transported to non-study hospitals, and we did not include them in this study.

This study has some limitations. First, it is unclear which if any of the severe TBI guideline recommendations are applicable to non-U.S. or non-European settings, especially since most of the data informing the TBI guidelines are not from Asia, Latin America or Africa, where TBI burden is high. This study is, therefore, particularly important because it examines the influence of some of these recommendations in a Latin American setting, and identification of hypoxia and poor nutrition as factors associated with poor outcomes appears to be relevant to both the US and Latin American contexts. Second, we do not have data for cohort beyond hospital discharge. Third, clinical care information from transferring hospitals was not collected as part of the parent study, thereby not allowing us to fully evaluate critical care after the immediately child was injured. We did not gather information on TBI education and cannot assess the role of ICU provider knowledge on TBI care or the number of reasons why children with TBI were transported by private vehicle rather than by ambulance. We also cannot discern decision making priorities and hierarchy between specialties in the ICU. We did not have access to data that allowed us to determine which clinical or radiographic signs of high ICP were used to exclude patients using sensitivity, specificity and predictive value of high ICP. We did not have post resuscitation GCS available for analysis but did use admission GCS motor scores which have previously been used in severe TBI studies. The purpose of this study was not to examine ICP and outcomes, and addition of this information would make this paper (focused on hypoxia and nutrition) diffuse. We cannot, therefore, address what percent use of hyperosmolar therapy is adequate or optimal. We used the 2012 Guidelines as a reference to examine clinical indicators for this study but not all guideline recommendations are within the scope of practice in Latin America. However, all the Latin American collaborators agreed that examining nutrition is important to TBI outcomes. Hence, the title of this paper and the discussion focus on specific recommendations such as hypoxia and nutrition. Finally, we agree that the level of evidence in the Guidelines is not level 1 but these are the best we currently have. Despite the aforementioned limitations, the present study provides new information on the current state of ICU pediatric TBI care in Argentina.

In summary, there is large variation in Argentine ICU practice in the care of children with TBI and second insults are common. Many children with evidence of high ICP do not receive ICP monitoring or hyperosmolar therapy. Yet, adherence to best practice TBI care by avoiding hypoxia and providing timely nutrition were associated with significantly favorable discharge outcomes. Implementing strategies that prevent hypoxia and facilitate early nutrition in the ICUs are urgently needed.

## Supporting information

S1 Table Clinical characteristics of 117 children with severe traumatic brain injury across seven study centers by discharge outcomes (univariate associations). (DOCX)
