# Supplementary material for: Intensive care treatments associated with favorable discharge outcomes in Argentine children with severe traumatic brain injury: For the South American Guideline Adherence Group
Source: PLoS One. 2017 Dec 15;12(12):e0189296. doi: 10.1371/journal.pone.0189296 (PMC5731744; doi:10.1371/journal.pone.0189296)
Supplement: S1 Table — (DOCX) [file pone.0189296.s001.docx]

**Supplemental Digital Content – S1 Table: Clinical Characteristics of 117 Children with Severe Traumatic Brain Injury across Seven Study Centers by Discharge Outcomes (Univariate Associations).**

|  | **Total** | ***favorable PCPC**** | ***Poor PCPC**** | ***P-value*** | ***favorable POPC***** | ***Poor POPC***** | ***P-value*** |
| --- | --- | --- | --- | --- | --- | --- | --- |
|  | ***n=117*** | ***n=95*** | ***n=22*** |  | ***n=96*** | ***n=21*** |  |
|  | **N(%)** | ***N(%)*** | ***N(%)*** |  | ***N(%)*** | ***N(%)*** |  |
| Age (years) mean[SD] | 8.8[4.6] | 9.0[4.7] | 8.0[4.0] | 0.381 | 9.0[4.7] | 8.1[4.1] | 0.237 |
|  |  |  |  |  |  |  |  |
| **Sex** |  |  |  | 0.7861 |  |  | 1.0 |
| Male | 78 (66.7) | 63 (66.3) | 15 (68.2) |  | 64 (66.7) | 14 (66.7) |  |
|  |  |  |  |  |  |  |  |
| **Injury mechanism** |  |  |  | 0.2473 |  |  | 0.2543 |
| Traffic accident | 68 (58.1) | 53 (55.8) | 15 (68.2) |  | 54 (56.3) | 14 (66.7) |  |
| Fall from height | 20 (17.1) | 18 (19.0) | 2 (9.1) |  | 18 (18.8) | 2 (9.5) |  |
| Fall from own height | 3 (2.6) | 3 (3.2) | 0 (0.0) |  | 3 (3.1) | 0 (0.0) |  |
| Strike | 11 (9.4) | 10 (10.5) | 1 (4.6) |  | 10 (10.4) | 1 (4.8) |  |
| Gunshot wound | 5 (4.3) | 2 (2.1) | 3 (13.6) |  | 2 (2.1) | 3 (14.3) |  |
| Other / Unknown | 10 (8.6) | 9 (9.5) | 1 (4.6) |  | 9 (9.4) | 1 (4.8) |  |
|  |  |  |  |  |  |  |  |
| **Injury circumstance** |  |  |  | 0.0939 |  |  | 0.1075 |
| Child abuse | 1 (0.9) | 0 (0.0) | 1 (4.6) |  | 0 (0.0) | 1 (4.8) |  |
| Intentional(no child abuse) | 4 (3.4) | 3 (3.2) | 1 (4.6) |  | 3 (3.1) | 1 (4.8) |  |
| Accidental | 111 (94.9) | 91 (95.8) | 20 (90.9) |  | 92 (95.8) | 19 (90.5) |  |
| Other / Unknown / Missing | 1 (0.9) | 1 (1.1) | 0 (0.0) |  | 1 (1.0) | 0 (0.0) |  |
|  |  |  |  |  |  |  |  |
| **Glasgow coma scale score (admit motor)** |  |  |  | 0.3827 |  |  | 0.3302 |
| 1 | 66 (56.4) | 52 (54.7) | 14 (63.6) |  | 53 (55.2) | 13 (61.9) |  |
| 2 | 7 (6.0) | 4 (4.2) | 3 (13.6) |  | 4 (4.2) | 3 (14.3) |  |
| 3 | 2 (1.7) | 2 (2.1) | 0 (0.0) |  | 2 (2.1) | 0 (0.0) |  |
| 4 | 18 (15.4) | 17 (17.9) | 4 (4.6) |  | 17 (17.7) | 1 (4.8) |  |
| 5 | 10 (8.6) | 9 (9.5) | 1 (4.6) |  | 9 (9.4) | 1 (4.8) |  |
| 6 | 3 (2.6) | 3 (3.2) | 0 (0.0) |  | 3 (3.1) | 0 (0.0) |  |
| Unknown | 11 (9.4) | 8 (8.4) | 3 (13.6) |  | 8 (8.3) | 3 (14.3) |  |
|  |  |  |  |  |  |  |  |
| **Head abbreviated injury severity score (AIS)** |  |  |  | 0.0717 |  |  | 0.0947 |
| 1 | 3 (2.6) | 3 (3.2) | 0 (0.0) |  | 3 (3.1) | 0 (0.0) |  |
| 2 | 21 (18.0) | 20 (21.1) | 1 (4.6) |  | 21 (21.9) | 0 90.0) |  |
| 3 | 33 (28.2) | 31 (32.6) | 2 (9.1) |  | 31 (32.3) | 2 (9.5) |  |
| 4 | 30 (25.6) | 25 (26.3) | 5 (22.7) |  | 24 (25.0) | 6 (28.6) |  |
| 5 | 29 (24.8) | 16 (16.8) | 13 (59.1) |  | 17 (17.7) | 12 (57.1) |  |
| 6 | 1 (0.9) | 0 (0.0) | 1 (4.6) |  | 0 (0.0) | 1 (4.8) |  |
|  |  |  |  |  |  |  |  |
| **Injury severity score** mean[SD] | 18.5[11.6] | 16.8[10.7] | 25.7[13.0] | **<0.001** | 16.8[10.6] | 30.0[13.1] | **<0.001** |
|  |  |  |  |  |  |  |  |
| **Non-head MAXAIS** |  |  |  | 0.6331 |  |  | 0.6457 |
| 0 | 55 (47.0) | 43 (45.3) | 12 (54.6) |  | 43 (44.8) | 12 (57.1) |  |
| 1 | 17 (14.5) | 15 (15.8) | 2 (9.1) |  | 15 (15.6) | 2 (9.5) |  |
| 2 | 18 (15.4) | 14 (14.7) | 4 (18.2) |  | 15 (15.6) | 3 (14.3) |  |
| 3 | 20 (17.1) | 16 (16.8) | 4 (18.2) |  | 16 (16.7) | 4 919.1) |  |
| 4 | 5 (4.3) | 5 (5.3) | 0 (0.0) |  | 5 (5.2) | 0 (0.0) |  |
| 5 | 2 (1.7) | 2 (2.1) | 0 (0.0) |  | 2 (2.1) | 0 (0.0) |  |
|  |  |  |  |  |  |  |  |
|  |  |  |  |  |  |  |  |
| **Hospital stay (days)** |  |  |  | **<0.001** |  |  | **<0.001** |
| mean[SD] | 20.0[18.7] | 16.4[14.4] | 35.9[26.0] |  | 16.4[14.4] | 36.5[26.5] |  |
|  |  |  |  |  |  |  |  |
| **Extracranial injury** |  |  |  | 0.5077 |  |  | **0.5983** |
| No | 108 (92.3) | 87 (91.6) | 21 (95.5) |  | 88 (91.7) | 20 (95.2) |  |
| Yes | 9 (7.7) | 8 (8.4) | 1 (4.6) |  | 8 (8.3) | 1 (4.8) |  |
|  |  |  |  |  |  |  |  |
| **Any surgery** |  |  |  | 0.2397 |  |  | 0.1501 |
| No | 80 (68.4) | 67 (70.5) | 13 (59.1) |  | 68 (70.8) | 12 (57.1) |  |
| Yes | 37 (31.6) | 28 (29.5) | 9 (40.9) |  | 28 (29.2) | 9 (42.9) |  |
|  |  |  |  |  |  |  |  |
| **Decompressive craniectomy** |  |  |  | **0.0338** |  |  | 0.0728 |
| No | 102 (87.2) | 87 (91.6) | 15 (68.2) |  | 87 (90.6) | 15 (71.4) |  |
| Yes | 14 (12.0) | 7 (7.4) | 7 (31.8) |  | 8 (8.3) | 6 (28.6) |  |
| NA / Missing | 1 (0.9) | 1 (1.0) | 0 (1.1) |  | 1 (1.0) | 0 (0.0) |  |

p values are corrected by adjusting clustering effect within trauma centers

*Dichotomous PCPC (poor outcome= severe-vegetative and death vs. favorable outcome = normal, mild-moderate disability)

**Dichotomous POPC (poor outcome = severe-vegetative state and death vs. favorable outcome = good-moderate overall performance)
